# On some binomial $B^{(m)}$-difference sequence spaces

**DOI:** 10.1186/s13660-017-1470-4

**Published:** 2017-08-22

**Authors:** Jian Meng, Meimei Song

**Affiliations:** Department of Mathematics, Tianjin University of Technology, Tianjin, 300000 P.R. China

**Keywords:** sequence space, matrix domain, Schauder basis, *α*-, *β*- and *γ*-duals

## Abstract

In this paper, we introduce the binomial sequence spaces $b^{a,b}_{0}(B^{(m)})$, $b^{a,b}_{c}(B^{(m)})$ and $b^{a,b}_{\infty}(B^{(m)})$ by combining the binomial transformation and difference operator. We prove the *BK*-property and some inclusion relations. Furthermore, we obtain Schauder bases and compute the *α*-, *β*- and *γ*-duals of these sequence spaces. Finally, we characterize matrix transformations on the sequence space $b_{c}^{a,b}(B^{(m)})$.

## Introduction and preliminaries

Let *w* denote the space of all sequences. By $\ell_{p}$, $\ell_{\infty }$, *c* and $c_{0}$, we denote the spaces of absolutely *p*-summable, bounded, convergent and null sequences, respectively, where $1\leq p<\infty$. A Banach sequence space *Z* is called a *BK*-space [1] provided each of the maps $p_{n}:Z\rightarrow\mathbb {C}$ defined by $p_{n}(x)=x_{n}$ is continuous for all $n\in\mathbb {N}$, which is of great importance in the characterization of matrix transformations between sequence spaces. One can prove that the sequence spaces $\ell_{\infty },c$ and $c_{0}$ are *BK*-spaces with their usual sup-norm.

Let *Z* be a sequence space, then Kizmaz [2] introduced the following difference sequence spaces:

$$\begin{aligned}& Z(\Delta)=\bigl\{ (x_{k})\in w:(\Delta x_{k})\in Z\bigr\} \end{aligned}$$

for $Z\in\{\ell_{\infty},c,c_{0}\}$, where $\Delta x_{k}=x_{k}-x_{k+1}$ for each $k\in\mathbb{N}$. Et and Colak [3] defined the generalization of the difference sequence spaces

$$\begin{aligned}& Z\bigl(\Delta^{m}\bigr)=\bigl\{ (x_{k})\in w:\bigl( \Delta^{m} x_{k}\bigr)\in Z\bigr\} \end{aligned}$$

for $Z\in\{\ell_{\infty},c,c_{0}\}$, where $m\in\mathbb{N}$, $\Delta ^{0}x_{k}=x_{k}$ and $\Delta^{m} x_{k}=\Delta^{m-1}x_{k}-\Delta ^{m-1}x_{k+1}$ for each $k\in\mathbb{N}$, which is equivalent to the binomial representation $\Delta^{m} x_{k}=\sum_{i=0}^{m}(-1)^{i}\bigl( {\scriptsize\begin{matrix}{}m \cr i\end{matrix}} \bigr) x_{k+i}$. Since then, many authors have studied further generalization of the difference sequence spaces [4–7]. Moreover, Altay and Polat [8], Başarir and Kara [9–13], Başarir, Kara and Konca [14], Kara [15], Kara and İlkhan [16, 17], Polat and Başar [18], Song and Meng [19] and many others have studied new sequence spaces from matrix point of view that represent difference operators.

For an infinite matrix $A=(a_{n,k})$ and $x=(x_{k})\in w$, the *A*-transform of *x* is defined by $Ax=\{(Ax)_{n}\}$ and is supposed to be convergent for all $n\in\mathbb{N}$, where $(Ax)_{n}=\sum_{k=0}^{\infty}a_{n,k}x_{k}$. For two sequence spaces *X* and *Y* and an infinite matrix $A=(a_{n,k})$, the sequence space $X_{A}$ is defined by

1.1 $$ X_{A}=\bigl\{ x=(x_{k})\in w:Ax \in X\bigr\} , $$

which is called the domain of matrix *A* in the space *X*. By $(X : Y)$, we denote the class of all matrices such that $X \subseteq Y_{A}$.

The Euler means $E^{r}$ of order *r* is defined by the matrix $E^{r}=(e_{n,k}^{r})$, where $0< r<1$ and

$$e_{n,k}^{r}= \textstyle\begin{cases} \bigl( {\scriptsize\begin{matrix}{} n\cr k \end{matrix}} \bigr)(1-r)^{n-k}r^{k}& \text{if $0\leq k\leq n$},\\ 0& \text{if $k>n$}. \end{cases} $$

The Euler sequence spaces $e^{r}_{0}$, $e^{r}_{c}$ and $e^{r}_{\infty}$ were defined by Altay and Başar [20] and Altay, Başar and Mursaleen [21] as follows:

$$\begin{aligned}& e^{r}_{0}=\left\{x=(x_{k})\in w: \lim _{n\rightarrow\infty}\sum_{k=0}^{n} \begin{pmatrix} n\\ k \end{pmatrix} (1-r)^{n-k}r^{k}x_{k}=0\right\}, \\& e^{r}_{c}=\left\{x=(x_{k})\in w: \lim _{n\rightarrow\infty}\sum_{k=0}^{n} \begin{pmatrix} n\\ k \end{pmatrix} (1-r)^{n-k}r^{k}x_{k} \text{ exists}\right\}, \end{aligned}$$

and

$$\begin{aligned}& e^{r}_{\infty}=\left\{x=(x_{k})\in w: \sup _{n\in\mathbb{N}}\left \vert \sum_{k=0}^{n} \left ( \begin{matrix} n\\ k \end{matrix} \right ) (1-r)^{n-k}r^{k}x_{k}\right \vert < \infty\right\}. \end{aligned}$$

Altay and Polat [8] defined further generalization of the Euler sequence spaces $e^{r}_{0}(\nabla)$, $e^{r}_{c}(\nabla)$ and $e^{r}_{\infty}(\nabla)$ by

$$Z (\nabla)=\bigl\{ x=(x_{k})\in w: (\nabla x_{k})\in Z \bigr\} $$

for $Z\in\{e_{0}^{r}, e_{c}^{r}, e_{\infty}^{r}\}$, where $\nabla x_{k}=x_{k}-x_{k-1}$ for each $k\in\mathbb{N}$. Here any term with negative subscript is equal to naught.

Polat and Başar [18] employed the matrix domain technique of the triangle limitation method for obtaining the following sequence spaces:

$$Z\bigl(\nabla^{(m)}\bigr)=\bigl\{ x=(x_{k})\in w: \bigl( \nabla^{(m)} x_{k}\bigr)\in Z\bigr\} $$

for $Z\in\{e_{0}^{r}, e_{c}^{r}, e_{\infty}^{r}\}$, where $\nabla ^{(m)}=(\delta_{n,k}^{(m)})$ is a triangle matrix defined by

$$\delta_{n,k}^{(m)}= \textstyle\begin{cases} (-1)^{n-k} \bigl( {\scriptsize\begin{matrix}{} m\cr n-k \end{matrix}} \bigr)& \text{if $\max\{0,n-m\}\leq k\leq n$},\\ 0& \text{if $0\leq k< \max\{0,n-m\}$ or $k>n$}, \end{cases} $$

for all $k,n,m\in\mathbb{N}$. Also, Başarir and Kayikçi [22] defined the matrix $B^{(m)}=(b_{n,k}^{(m)})$ by

$$b_{n,k}^{(m)}= \textstyle\begin{cases} \bigl( {\scriptsize\begin{matrix}{} m\cr n-k \end{matrix}} \bigr)r^{m-n+k}s^{n-k}& \text{if $\max\{0,n-m\}\leq k\leq n$},\\ 0& \text{if $0\leq k< \max\{0,n-m\}$ or $k>n$}, \end{cases} $$

which is reduced to the matrix $\nabla^{(m)}$ in the case $r=1$, $s=-1$. Kara and Başarir [23] introduced the spaces $e^{r}_{0}(B^{(m)})$, $e^{r}_{c}(B^{(m)})$ and $e^{r}_{\infty }(B^{(m)})$ of $B^{(m)}$-difference sequences.

Recently Bişgin [24, 25] defined another generalization of the Euler sequence spaces and introduced the binomial sequence spaces $b^{a,b}_{0}$, $b^{a,b}_{c}$, $b^{a,b}_{\infty}$ and $b^{a,b}_{p}$. Let $a,b\in\mathbb{R}$ and $a,b\neq0$. Then the binomial matrix $B^{a,b}=(b_{n,k}^{a,b})$ is defined by

$$b_{n,k}^{a,b}= \textstyle\begin{cases} \frac{1}{(a+b)^{n}}\bigl( {\scriptsize\begin{matrix}{} n\cr k \end{matrix}} \bigr)a^{n-k}b^{k}& \text{if $0\leq k\leq n$},\\ 0& \text{if $k>n$}, \end{cases} $$

for all $k,n\in\mathbb{N}$. For $ab>0$ we have

(i) $\Vert B^{a,b}\Vert <\infty$,
(ii) $\lim_{n\rightarrow\infty}b_{n,k}^{a,b}=0$ for each $k\in \mathbb{N}$,
(iii) $\lim_{n\rightarrow\infty}\sum_{k}b_{n,k}^{a,b}=1$.

Thus, the binomial matrix $B^{a,b}$ is regular for $ab>0$. Unless stated otherwise, we assume that $ab >0$. If we take $a+b =1$, we obtain the Euler matrix $E^{r}$, so the binomial matrix generalizes the Euler matrix. Bişgin defined the following binomial sequence spaces:

$$\begin{aligned}& b^{a,b}_{0}=\left\{x=(x_{k})\in w: \lim _{n\rightarrow\infty}\frac {1}{(a+b)^{n}}\sum_{k=0}^{n} \left ( \begin{matrix} n\\ k \end{matrix} \right )a^{n-k}b^{k}x_{k}=0\right\}, \\& b^{a,b}_{c}=\left\{x=(x_{k})\in w: \lim _{n\rightarrow\infty}\frac {1}{(a+b)^{n}}\sum_{k=0}^{n} \left ( \begin{matrix} n\\ k \end{matrix} \right )a^{n-k}b^{k}x_{k} \text{ exists}\right\}, \end{aligned}$$

and

$$\begin{aligned}& b^{a,b}_{\infty}=\left\{x=(x_{k})\in w: \sup _{n\in\mathbb{N}}\left \vert \frac {1}{(a+b)^{n}}\sum _{k=0}^{n}\left ( \begin{matrix} n\\ k \end{matrix} \right )a^{n-k}b^{k}x_{k} \right \vert < \infty\right\}. \end{aligned}$$

The purpose of the present paper is to study the binomial difference spaces $b^{a,b}_{0}(B^{(m)})$, $b^{a,b}_{c}(B^{(m)})$ and $b^{a,b}_{\infty}(B^{(m)})$ whose $B^{a,b}(B^{(m)})$-transforms are in the spaces $c_{0}$, *c* and $\ell_{\infty}$, respectively. These new sequence spaces are the generalization of the sequence spaces defined in [24, 25] and [23]. Also, we give some inclusion relations and compute the bases and *α*-, *β*- and *γ*-duals of these sequence spaces.

## The binomial difference sequence spaces

In this section, we introduce the spaces $b^{a,b}_{0}(B^{(m)})$, $b^{a,b}_{c}(B^{(m)})$ and $b^{a,b}_{\infty}(B^{(m)})$ and prove the *BK*-property and inclusion relations.

We first define the binomial difference sequence spaces $b^{a,b}_{0}(B^{(m)})$, $b^{a,b}_{c}(B^{(m)})$ and $b^{a,b}_{\infty }(B^{(m)})$ by

$$\begin{aligned}& Z\bigl(B^{(m)}\bigr)=\bigl\{ x=(x_{k})\in w: \bigl(B^{(m)} x_{k}\bigr)\in Z\bigr\} \end{aligned}$$

for $Z\in\{b^{a,b}_{0}, b^{a,b}_{c}, b^{a,b}_{\infty}\}$. By using the notion of (1.1), the sequence spaces $b^{a,b}_{0}(B^{(m)})$, $b^{a,b}_{c}(B^{(m)})$ and $b^{a,b}_{\infty }(B^{(m)})$ can be redefined by

2.1 $$ b^{a,b}_{0}\bigl(B^{(m)}\bigr)= \bigl(b^{a,b}_{0}\bigr)_{B^{(m)}}, \qquad b^{a,b}_{c} \bigl(B^{(m)}\bigr)=\bigl(b^{a,b}_{c} \bigr)_{B^{(m)}},\qquad b^{a,b}_{\infty }\bigl(B^{(m)}\bigr)= \bigl(b^{a,b}_{\infty}\bigr)_{B^{(m)}}. $$

It is obvious that the sequence spaces $b^{a,b}_{0}(B^{(m)})$, $b^{a,b}_{c}(B^{(m)})$ and $b^{a,b}_{\infty}(B^{(m)})$ may be reduced to some sequence spaces in the special cases of $a, b, s, r$ and $m\in\mathbb{N}$. For instance, if we take $a+b=1$, then we obtain the spaces $e^{r}_{0}(B^{(m)}) $, $e^{r}_{c}(B^{(m)})$ and $e^{r}_{\infty}(B^{(m)}) $, defined by Kara and Başarir [23]. If we take $a+b=1$, $r=1$ and $s=-1$, then we obtain the spaces $e^{r}_{0}(\nabla^{(m)}), e^{r}_{c}(\nabla^{(m)})$ and $e^{r}_{\infty}(\nabla^{(m)})$, defined by Polat and Başar [18]. Especially, taking $r=1$ and $s=-1$, we obtain the new binomial difference sequence spaces $b^{a,b}_{0}(\nabla^{(m)}), b^{a,b}_{c}(\nabla^{(m)})$ and $b^{a,b}_{\infty}(\nabla^{(m)})$.

Let us define the sequence $y=(y_{n})$ as the $B^{a,b}(B^{(m)})$-transform of a sequence $x=(x_{k})$, that is,

2.2 $$ y_{n}=\bigl[B^{a,b}\bigl(B^{(m)} x_{k}\bigr)\bigr]_{n} =\frac{1}{(a+b)^{n}}\sum _{k=0}^{n}\sum_{i=k}^{n} \left ( \begin{matrix} m\\ i-k \end{matrix} \right )\left ( \begin{matrix} n\\ i \end{matrix} \right )a^{n-i}b^{i}r^{m+k-i}s^{i-k}x_{k} $$

for each $n\in\mathbb{N}$. Then the sequence spaces $b^{a,b}_{0}(B^{(m)})$, $b^{a,b}_{c}(B^{(m)})$ and $b^{a,b}_{\infty }(B^{(m)})$ can be redefined by all sequences whose $B^{a,b}(B^{(m)})$-transforms are in the spaces $c_{0}$, *c* and $\ell _{\infty}$.

### Theorem 2.1

*The sequence spaces* $b^{a,b}_{0}(B^{(m)})$, $b^{a,b}_{c}(B^{(m)})$ *and* $b^{a,b}_{\infty}(B^{(m)})$ *are* *BK*-*spaces with their sup*-*norm defined by*

$$\begin{aligned}& \Vert x\Vert _{b^{a,b}_{0}(B^{(m)})}=\Vert x\Vert _{b^{a,b}_{c}(B^{(m)})}=\Vert x \Vert _{b^{a,b}_{\infty }(B^{(m)})}=\sup_{n\in\mathbb{N}}\bigl\vert \bigl[B^{a,b}\bigl(B^{(m)} x_{k}\bigr) \bigr]_{n}\bigr\vert . \end{aligned}$$

### Proof

The sequence spaces $b^{a,b}_{0}$, $b^{a,b}_{c}$ and $b^{a,b}_{\infty}$ are *BK*-spaces with their sup-norm (see [24], Theorem 2.1 and [25], Theorem 2.1). Moreover, $B^{(m)}$ is a triangle matrix and (2.1) holds. By using Theorem 4.3.12 of Wilansky [26], we deduce that the binomial sequence spaces $b^{a,b}_{0}(B^{(m)})$, $b^{a,b}_{c}(B^{(m)})$ and $b^{a,b}_{\infty}(B^{(m)})$ are *BK*-spaces. □

### Theorem 2.2

*The sequence spaces* $b^{a,b}_{0}(B^{(m)})$, $b^{a,b}_{c}(B^{(m)})$ *and* $b^{a,b}_{\infty}(B^{(m)})$ *are linearly isomorphic to the spaces* $c_{0}$, *c* *and* $\ell_{\infty}$, *respectively*.

### Proof

Similarly, we prove the theorem only for the space $b^{a,b}_{0}(B^{(m)})$. To prove $b^{a,b}_{0}(B^{(m)})\cong c_{0}$, we must show the existence of a linear bijection between the spaces $b^{a,b}_{0}(B^{(m)})$ and $c_{0}$.

Consider $b^{a,b}_{0}(B^{(m)})\rightarrow c_{0}$ by $T(x)=B^{a,b}(B^{(m)} x_{k})$. The linearity of *T* is obvious and $x=0$ whenever $T(x)=0$. Therefore, *T* is injective.

Let $y=(y_{n})\in c_{0} $ and define the sequence $x=(x_{k})$ by

2.3 $$ x_{k}=\sum_{i=0}^{k}(a+b)^{i} \sum_{j=i}^{k}\left ( \begin{matrix} m+k-j-1\\ k-j \end{matrix} \right ) \left ( \begin{matrix} j\\ i \end{matrix} \right )\frac{(-s)^{k-j}}{r^{m+k-j}}(-a)^{j-i}b^{-j}y_{i} $$

for each $k \in\mathbb{N}$. Then we have

$$\lim_{n\rightarrow\infty}\bigl[B^{a,b}\bigl(B^{(m)} x_{k}\bigr)\bigr]_{n}=\lim_{n\rightarrow \infty} \frac{1}{(a+b)^{n}}\sum_{k=0}^{n}\left ( \begin{matrix} n\\ k \end{matrix} \right )a^{n-k}b^{k} \bigl(B^{(m)} x_{k}\bigr)=\lim_{n\rightarrow\infty}y_{n}=0, $$

which implies that $x\in b^{a,b}_{0}(B^{(m)})$ and $T(x)=y$. Consequently, *T* is surjective and is norm preserving. Thus, $b^{a,b}_{0}(B^{(m)})\cong c_{0}$. □

The following theorems give some inclusion relations for this class of sequence spaces. We have the well known inclusion $c_{0}\subseteq c\subseteq\ell_{\infty}$, then the corresponding extended versions also preserve this inclusion.

### Theorem 2.3

*The inclusion* $b^{a,b}_{0}(B^{(m)})\subseteq b^{a,b}_{c}(B^{(m)})\subseteq b^{a,b}_{\infty}(B^{(m)})$ *holds*.

### Theorem 2.4

*The inclusions* $e_{0}^{a}(B^{(m)})\subseteq b^{a,b}_{0}(B^{(m)})$, $e_{c}^{a}(B^{(m)})\subseteq b^{a,b}_{c}(B^{(m)})$ *and* $e_{\infty }^{a}(B^{(m)})\subseteq b^{a,b}_{\infty}(B^{(m)})$ *strictly hold*.

### Proof

Similarly, we only prove the inclusion $e_{0}^{a}(B^{(m)})\subseteq b^{a,b}_{0}(B^{(m)})$. If $a+b=1$, we have $E^{a}=B^{a,b}$. So $e_{0}^{a}(B^{(m)})\subseteq b^{a,b}_{0}(B^{(m)})$ holds. Let $0< a<1$ and $b=4$. We define a sequence $x=(x_{k})$ by $x_{k}=(-\frac {3}{a})^{k}$ for each $k\in\mathbb{N}$. It is clear that

$$E^{a}\bigl(B^{(m)} x_{k}\bigr)=\left(\sum _{i=0}^{m}\left ( \begin{matrix} m\\ i \end{matrix} \right )s^{i}r^{m-i} \biggl(-\frac{a}{3}\biggr)^{i}(-2-a)^{n}\right)\notin c_{0} $$

and

$$B^{a,b}\bigl(B^{(m)} x_{k}\bigr) =\left(\sum _{i=0}^{m}\left ( \begin{matrix} m\\ i \end{matrix} \right )s^{i}r^{m-i} \biggl(-\frac{a}{3}\biggr)^{i}\biggl(\frac{1}{4+a} \biggr)^{n}\right)\in c_{0}. $$

So, we have $x\in b^{a,b}_{0}(B^{(m)})\setminus e_{0}^{a}(B^{(m)})$. This shows that the inclusion $e_{0}^{a}(B^{(m)})\subseteq b^{a,b}_{0}(B^{(m)})$ strictly holds. □

## The Schauder basis and *α*-, *β*- and *γ*-duals

For a normed space $(X, \Vert \cdot\Vert )$, a sequence $\{ x_{k}:x_{k}\in X\}_{k\in\mathbb{N}}$ is called a $Schauder$ $basis$ [1] if for every $x\in X$, there is a unique scalar sequence $(\lambda_{k})$ such that $\Vert x-\sum_{k=0}^{n}\lambda _{k}x_{k}\Vert \rightarrow0$ as $n\rightarrow\infty$. We shall construct Schauder bases for the sequence spaces $b_{0}^{a,b}(B^{(m)})$ and $b_{c}^{a,b}(B^{(m)})$.

We define the sequence $g^{(k)}(a,b)=\{g^{(k)}_{i}(a,b)\}_{i \in\mathbb {N}}$ by

$$g^{(k)}_{i}(a,b)= \textstyle\begin{cases} 0& \text{if $0\leq i < k$},\\ (a+b)^{k}\sum_{j=k}^{i}\bigl( {\scriptsize\begin{matrix}{} m+i-j-1\\ i-j \end{matrix}} \bigr)\bigl( {\scriptsize\begin{matrix}{} j\cr k \end{matrix}} \bigr)\frac{(-s)^{i-j}}{r^{m+i-j}}b^{-j}(-a)^{j-k}& \text{if $i\geq k$}, \end{cases} $$

for each $k\in\mathbb{N}$.

### Theorem 3.1

*The sequence* $(g^{(k)}(a,b))_{k\in\mathbb{N}}$ *is a Schauder basis for the binomial sequence space* $b_{0}^{a,b}(B^{(m)})$ *and every* $x=(x_{i})\in b_{0}^{a,b}(B^{(m)})$ *has a unique representation by*

3.1 $$ x=\sum_{k} \lambda_{k}(a,b) g^{(k)}(a,b), $$

*where* $\lambda_{k}(a,b)= [B^{a,b}(B^{(m)} x_{i})]_{k}$ *for each* $k\in \mathbb{N}$.

### Proof

Obviously, $B^{a,b}(B^{(m)} g^{(k)}_{i}(a,b))=e_{k}\in c_{0}$, where $e_{k}$ is the sequence with 1 in the *k*th place and zeros elsewhere for each $k\in\mathbb{N}$. This implies that $g^{(k)}(a,b)\in b_{0}^{a,b}(B^{(m)})$ for each $k\in\mathbb{N}$.

For $x \in b_{0}^{a,b}(B^{(m)})$ and $n\in\mathbb{N}$, we put

$$x^{(n)}=\sum_{k=0}^{n} \lambda_{k}(a,b) g^{(k)}(a,b). $$

By the linearity of $B^{a,b}(B^{(m)})$, we have

$$B^{a,b}\bigl(B^{(m)} x^{(n)}_{i}\bigr)=\sum _{k=0}^{n}\lambda _{k}(a,b)B^{a,b} \bigl(B^{(m)} g^{(k)}_{i}(a,b)\bigr)=\sum _{k=0}^{n}\lambda_{k}(a,b)e_{k} $$

and

$$\bigl[B^{a,b}\bigl(B^{(m)}\bigl(x_{i}-x_{i}^{(n)} \bigr)\bigr)\bigr]_{k}= \textstyle\begin{cases} 0& \text{if $0\leq k < n$},\\ [B^{a,b}(B^{(m)} x_{i})]_{k}& \text{if $k\geq n$}, \end{cases} $$

for each $k\in\mathbb{N}$.

For every $\varepsilon>0$, there is a positive integer $n_{0}$ such that

$$\bigl\vert \bigl[B^{a,b}\bigl(B^{(m)} x_{i}\bigr) \bigr]_{k}\bigr\vert < \frac{\varepsilon}{2} $$

for all $k\geq n_{0}$. Then we have

$$\bigl\Vert x-x^{(n)}\bigr\Vert _{b_{0}^{a,b}(B^{(m)})}=\sup _{k\geq n}\bigl\vert \bigl[B^{a,b}\bigl(B^{(m)} x_{i}\bigr)\bigr]_{k}\bigr\vert \leq\sup _{k\geq n_{0}}\bigl\vert \bigl[B^{a,b}\bigl(B^{(m)} x_{i}\bigr)\bigr]_{k}\bigr\vert < \frac{\varepsilon}{2}< \varepsilon, $$

which implies that $x \in b_{0}^{a,b}(B^{(m)})$ is represented as in (3.1).

To show the uniqueness of this representation, we assume that

$$x=\sum_{k} \mu_{k}(a,b) g^{(k)}(a,b). $$

Then we have

$$\bigl[B^{a,b}\bigl(B^{(m)} x_{i}\bigr) \bigr]_{k}=\sum_{k}\mu_{k}(a,b) \bigl[B^{a,b}\bigl(B^{(m)} g^{(k)}_{i}(a,b) \bigr)\bigr]_{k}=\sum_{k} \mu_{k}(a,b) (e_{k})_{k}=\mu_{k}(a,b), $$

which is a contradiction with the assumption that $\lambda _{k}(a,b)=[B^{a,b}(B^{(m)} x_{i})]_{k}$ for each $k \in\mathbb{N}$. This shows the uniqueness of this representation. □

### Theorem 3.2

*Let* $g=(1,1,1,1,\ldots)$ *and* $lim_{k\rightarrow\infty}\lambda_{k}(a,b)=l$. *The set* $\{g, g^{(0)}(a,b), g^{(1)}(a,b),\ldots, g^{(k)}(a,b),\ldots\}$ *is a Schauder basis for the space* $b_{c}^{a,b}(B^{(m)})$ *and every* $x\in b_{c}^{a,b}(B^{(m)})$ *has a unique representation by*

3.2 $$ x=lg+\sum_{k} \bigl[ \lambda_{k}(a,b)-l\bigr] g^{(k)}(a,b). $$

### Proof

Obviously, $B^{a,b}(B^{(m)} g^{k}_{i}(a,b))=e_{k}\in c$ and $g\in b_{c}^{a,b}(B^{(m)})$. For $x \in b_{c}^{a,b}(B^{(m)})$, we put $y=x-lg$ and we have $y\in b_{0}^{a,b}(B^{(m)})$. Hence, we deduce that *y* has a unique representation by (3.1), which implies that *x* has a unique representation by (3.2). Thus, we complete the proof. □

### Corollary 3.3

*The sequence spaces* $b_{0}^{a,b}(B^{(m)})$ *and* $b_{c}^{a,b}(B^{(m)})$ *are separable*.

Köthe and Toeplitz [27] first computed the dual whose elements can be represented as sequences and defined the *α*-dual (or Köthe-Toeplitz dual). Next, we compute the *α*-,*β*- and *γ*-duals of the sequence spaces $b_{0}^{a,b}(B^{(m)})$, $b_{c}^{a,b}(B^{(m)})$ and $b_{\infty }^{a,b}(B^{(m)})$.

For the sequence spaces *X* and *Y*, define multiplier space $M(X,Y)$ by

$$M(X,Y)=\bigl\{ u=(u_{k})\in w:ux=(u_{k}x_{k})\in Y \text{ for all } x=(x_{k})\in X\bigr\} . $$

Then the *α*-, *β*- and *γ*-duals of a sequence space *X* are defined by

$$X^{\alpha}=M(X,\ell_{1}), \qquad X^{\beta}=M(X,c) \quad \mbox{and}\quad X^{\gamma }=M(X,\ell_{\infty}), $$

respectively.

Let us give the following properties:

3.3 $$\begin{aligned}& \sup_{K\in\Gamma} \sum_{n} \biggl\vert \sum_{k\in K} a_{n,k}\biggr\vert < \infty, \end{aligned}$$

3.4 $$\begin{aligned}& \sup_{n\in\mathbb{N}} \sum_{k} \vert a_{n,k}\vert < \infty, \end{aligned}$$

3.5 $$\begin{aligned}& \lim_{n\rightarrow\infty}a_{n,k}=a_{k} \quad \text{for each } k\in \mathbb{N}, \end{aligned}$$

3.6 $$\begin{aligned}& \lim_{n\rightarrow\infty}\sum_{k}a_{n,k}=a, \end{aligned}$$

3.7 $$\begin{aligned}& \lim_{n\rightarrow\infty}\sum_{k} \vert a_{n,k}\vert =\sum_{k}\Bigl\vert \lim_{n\rightarrow\infty}a_{n,k}\Bigr\vert , \end{aligned}$$

where Γ is the collection of all finite subsets of $\mathbb{N}$.

### Lemma 3.4

[28]

*Let* $A=(a_{n,k})$ *be an infinite matrix*. *Then the following statements hold*:

(i) $A\in(c_{0}:\ell_{1})=(c:\ell_{1})=(\ell_{\infty}:\ell_{1})$ *if and only if* (3.3) *holds*.
(ii) $A\in(c_{0}:c)$ *if and only if* (3.4) *and* (3.5) *hold*.
(iii) $A\in(c:c)$ *if and only if* (3.4), (3.5) *and* (3.6) *hold*.
(iv) $A\in(\ell_{\infty}:c)$ *if and only if* (3.5) *and* (3.7) *hold*.
(v) $A\in(c_{0}:\ell_{\infty})=(c:\ell_{\infty})=(\ell_{\infty}:\ell _{\infty})$ *if and only if* (3.4) *holds*.

### Theorem 3.5

*The* *α*-*dual of the spaces* $b_{0}^{a,b}(B^{(m)})$, $b_{c}^{a,b}(B^{(m)})$ *and* $b_{\infty}^{a,b}(B^{(m)})$ *is the set*

$$\begin{aligned} U^{a,b}_{1} =& \Biggl\{ u=(u_{k})\in w: \sup _{K\in\Gamma}\sum_{k}\Biggl\vert \sum _{i\in K} (a+b)^{i}\sum _{j=i}^{k}\left ( \begin{matrix} m+k-j-1\\ k-j \end{matrix} \right )\left ( \begin{matrix} j\\ i \end{matrix} \right ) \\ \phantom{\sum _{j=i}^{k}\begin{matrix} j\\ i \end{matrix}} & {}\times \frac{(-s)^{k-j}}{r^{m+k-j}}b^{-j}(-a)^{j-i}u_{k} \Biggr\vert < \infty\Biggr\} . \end{aligned}$$

### Proof

Let $u=(u_{k})\in w$ and $x=(x_{k})$ be defined by (2.3), then we have

$$\begin{aligned}& u_{k}x_{k}=\sum_{i=0}^{k}(a+b)^{i} \sum_{j=i}^{k}\left ( \begin{matrix} m+k-j-1\\ k-j \end{matrix} \right )\left ( \begin{matrix} j\\ i \end{matrix} \right )\frac{(-s)^{k-j}}{r^{m+k-j}}b^{-j}(-a)^{j-i}u_{k}y_{i}= \bigl(G^{a,b}y\bigr)_{k} \end{aligned}$$

for each $k\in\mathbb{N}$, where $G^{a,b}=(g^{a,b}_{k,i})$ is defined by

$$g^{a,b}_{k,i}= \textstyle\begin{cases} (a+b)^{i}\sum_{j=i}^{k}\bigl( {\scriptsize\begin{matrix}{} m+k-j-1\cr k-j \end{matrix}} \bigr)\bigl( {\scriptsize\begin{matrix}{} j\cr i \end{matrix}} \bigr)\frac{(-s)^{k-j}}{r^{m+k-j}}b^{-j}(-a)^{j-i}u_{k}& \text{if $0\leq i\leq k$},\\ 0& \text{if $i>k$}. \end{cases} $$

Therefore, we deduce that $ux= (u_{k}x_{k})\in\ell_{1}$ whenever $x\in b_{0}^{a,b}(B^{(m)})$, $b_{c}^{a,b}(B^{(m)})$ or $b_{\infty}^{a,b}(B^{(m)})$, if and only if $G^{a,b}y\in\ell_{1}$, whenever $y\in c_{0}, c$ or $\ell_{\infty}$. This implies that $u=(u_{k})\in [b_{0}^{a,b}(B^{(m)})]^{\alpha}, [b_{c}^{a,b}(B^{(m)})]^{\alpha}$ or $[b_{\infty}^{a,b}(B^{(m)})]^{\alpha}$ if and only if $G^{a,b}\in (c_{0}:\ell_{1})$, $G^{a,b}\in(c:\ell_{1})$ or $G^{a,b}\in(\ell_{\infty }:\ell_{1})$ by Parts (i) of Lemma 3.4. So we obtain

$$u=(u_{k})\in\bigl[b_{0}^{a,b}\bigl(B^{(m)} \bigr)\bigr]^{\alpha }=\bigl[b_{c}^{a,b} \bigl(B^{(m)}\bigr)\bigr]^{\alpha} =\bigl[b_{\infty}^{a,b} \bigl(B^{(m)}\bigr)\bigr]^{\alpha} $$

if and only if

$$\sup_{K\in\Gamma}\sum_{k}\left \vert \sum_{i\in K}(a+b)^{i}\sum _{j=i}^{k}\left ( \begin{matrix} m+k-j-1\\ k-j \end{matrix} \right )\left ( \begin{matrix} j\\ i \end{matrix} \right )\frac{(-s)^{k-j}}{r^{m+k-j}}b^{-j}(-a)^{j-i}u_{k} \right \vert < \infty. $$

Thus, we have $[b_{0}^{a,b}(B^{(m)})]^{\alpha }=[b_{c}^{a,b}(B^{(m)})]^{\alpha} =[b_{\infty}^{a,b}(B^{(m)})]^{\alpha }=U^{a,b}_{1}$. □

Now, we define the sets $U_{2}^{a,b}$, $U_{3}^{a,b}$, $U_{4}^{a,b}$ and $U_{5}^{a,b}$ by

$$\begin{aligned}& U_{2}^{a,b}=\biggl\{ u=(u_{k})\in w: \sup _{n\in\mathbb{N}}\sum_{k}\vert u_{n,k}\vert < \infty\biggr\} , \\& U_{3}^{a,b}=\Bigl\{ u=(u_{k})\in w: \lim _{n\rightarrow\infty} u_{n,k} \text{ exists for each } k \in\mathbb{N} \Bigr\} , \\& U_{4}^{a,b}=\biggl\{ u=(u_{k})\in w: \lim _{n\rightarrow\infty}\sum_{k}\vert u_{n,k}\vert =\sum_{k}\Bigl\vert \lim _{n\rightarrow\infty}u_{n,k}\Bigr\vert \biggr\} , \end{aligned}$$

and

$$\begin{aligned}& U_{5}^{a,b}=\biggl\{ u=(u_{k})\in w: \lim _{n\rightarrow\infty}\sum_{k}u_{n,k} \text{ exists}\biggr\} , \end{aligned}$$

where

$$\begin{aligned} u_{n,k}=(a+b)^{k}\sum_{i=k}^{n} \sum_{j=k}^{i}\left ( \begin{matrix} m+i-j-1\\ i-j \end{matrix} \right )\left ( \begin{matrix} j\\ k \end{matrix} \right )\frac{(-s)^{i-j}}{r^{m+i-j}}(-a)^{j-k}(b)^{-j}u_{i}. \end{aligned}$$

### Theorem 3.6

*We have the following relations*:

(i) $[b_{0}^{a,b}(B^{(m)})]^{ \beta}=U_{2}^{a,b}\cap U_{3}^{a,b}$,
(ii) $[b_{c}^{a,b}(B^{(m)})]^{ \beta}=U_{2}^{a,b}\cap U_{3}^{a,b}\cap U_{5}^{a,b}$,
(iii) $[b_{\infty}^{a,b}(B^{(m)})]^{ \beta}=U_{3}^{a,b}\cap U_{4}^{a,b}$.

### Proof

Let $u=(u_{k})\in w$ and $x=(x_{k})$ be defined by (2.3), then we consider the following equation:

$$\begin{aligned} \sum_{k=0}^{n}u_{k}x_{k} =& \sum_{k=0}^{n}u_{k}\left[\sum _{i=0}^{k}(a+b)^{i}\sum _{j=i}^{k}\left ( \begin{matrix} m+k-j-1\\ k-j \end{matrix} \right ) \left ( \begin{matrix} j\\ i \end{matrix} \right )\frac{(-s)^{k-j}}{r^{m+k-j}}(-a)^{j-i}b^{-j}y_{i} \right] \\ =&\sum_{k=0}^{n}\left[(a+b)^{k} \sum_{i=k}^{n}\sum _{j=k}^{i}\left ( \begin{matrix} m+i-j-1\\ i-j \end{matrix} \right )\left ( \begin{matrix} j\\ k \end{matrix} \right )\frac{(-s)^{i-j}}{r^{m+i-j}}(-a)^{j-k}b^{-j}u_{i} \right]y_{k} \\ =&\bigl(U^{a,b}y\bigr)_{n}, \end{aligned}$$

where $U^{a,b}=(u^{a,b}_{n,k})$ is defined by

$$u_{n,k}^{a,b}= \textstyle\begin{cases} (a+b)^{k}\sum_{i=k}^{n}\sum_{j=k}^{i}\bigl( {\scriptsize\begin{matrix}{} m+i-j-1\cr i-j \end{matrix}} \bigr)\bigl( {\scriptsize\begin{matrix}{} j\cr k \end{matrix}} \bigr)\frac{(-s)^{i-j}}{r^{m+i-j}}(-a)^{j-k}(b)^{-j}u_{i}& \text{if $0\leq k \leq n$},\\ 0& \text{if $k> n$}. \end{cases} $$

Therefore, we deduce that $ux= (u_{k}x_{k})\in c$ whenever $x\in b_{0}^{a,b}(B^{(m)})$ if and only if $U^{a,b}y\in c$ whenever $y\in c_{0}$, which implies that $u=(u_{k})\in[b_{0}^{a,b}(B^{(m)})]^{ \beta}$ if and only if $U^{a,b}\in(c_{0}:c)$ by Part (ii) of Lemma 3.4. So we obtain $[b_{0}^{a,b}(B^{(m)})]^{ \beta}=U_{2}^{a,b}\cap U_{3}^{a,b}$. Using Parts (iii), (iv) instead of Part (ii) of Lemma 3.4, the proof can be completed in a similar way. □

Similarly, we give the following theorem without proof.

### Theorem 3.7

*The* *γ*-*dual of the spaces* $b_{0}^{a,b}(B^{(m)})$, $b_{c}^{a,b}(B^{(m)})$ *and* $b_{\infty}^{a,b}(B^{(m)})$ *is the set* $U_{2}^{a,b}$.

## Certain matrix mappings on the space $b_{c}^{a,b}(B^{(m)})$

In this section, we characterize matrix transformations from $b_{c}^{a,b}(B^{(m)})$ into $\ell_{p}$, $\ell_{\infty}$ and *c*. Let us define the matrix $\Theta=(\theta_{n,k})$ via an infinite matrix $\Lambda=(\lambda_{n,k})$ by $\Theta=\Lambda(B^{a,b}(B^{(m)}))^{-1}$, that is,

4.1 $$ \theta_{n,k}=(a+b)^{k}\sum _{j=k}^{\infty} \left ( \begin{matrix} m+n-j-1\\ n-j \end{matrix} \right ) \left ( \begin{matrix} j\\ k \end{matrix} \right )\frac{(-s)^{n-j}}{r^{m+n-j}}(-a)^{j-k}b^{-j} \lambda_{n,j}, $$

where $(B^{a,b}(B^{(m)}))^{-1}$ is the inverse of the $B^{a,b}(B^{(m)})$-transform. We now give the following lemmas.

### Lemma 4.1

*Let* *Z* *be any given sequence space and the entries of the matrices* $\Lambda=(\lambda_{n,k})$ *and* $\Theta=(\theta_{n,k})$ *are connected with equation* (4.1). *If* $(\lambda_{n,k})_{k}\in [b_{c}^{a,b}(B^{(m)})]^{ \beta}$ *for all* $n\in\mathbb{N}$, *then* $\Lambda\in(b_{c}^{a,b}(B^{(m)}): Z)$ *if and only if* $\Theta\in(c:Z)$.

### Proof

Let $\Lambda\in(b_{c}^{a,b}(B^{(m)}): Z)$ and $y=(y_{n})\in c$. For every $x=(x_{k})\in b_{c}^{a,b}(B^{(m)})$, we have $x_{k}=[(B^{a,b}(B^{(m)}))^{-1}y_{n}]_{k}$. Since $(\lambda _{n,k})_{k}\in[b_{c}^{a,b}(B^{(m)})]^{ \beta}$ for all $n\in\mathbb {N}$, this implies the existence of the Λ-transform of *x*, *i.e.* Λ*x* exists. So we obtain $\Lambda x=\Lambda (B^{a,b}(B^{(m)}))^{-1}y=\Theta y$, which implies that $\Theta\in(c:Z)$.

Conversely, let $\Theta\in(c:Z)$ and $x\in b_{c}^{a,b}(B^{(m)})$. For every $y\in c$, we have $y_{n}=[B^{a,b}(B^{(m)}x_{k})]_{n}$. Since $(\lambda_{n,k})_{k}\in[b_{c}^{a,b}(B^{(m)})]^{ \beta}$ for all $n\in \mathbb{N}$, this implies that Θ*y* exists, which can be proved in a similar way to the proof of Theorem 3.6. So we have $\Theta y=\Theta B^{a,b}(B^{(m)})x=\Lambda x$, which shows that $\Lambda\in (b_{c}^{a,b}(B^{(m)}):Z)$. □

### Lemma 4.2

[28]

*Let* $A=(a_{n,k})$ *be an infinite matrix*. *Then the following statement holds*: $A\in(c:\ell_{p})$ *if and only if*

4.2 $$ \sup_{K\in\Gamma}\sum_{n} \biggl\vert \sum_{k\in K}a_{n,k}\biggr\vert ^{p}< \infty,\quad 1\leq p< \infty. $$

For brevity of notation, we write

$$\begin{aligned}& t_{n,k}=(a+b)^{k}\sum_{i=k}^{n} \sum_{j=k}^{i}\left ( \begin{matrix} m+i-j-1\\ i-j \end{matrix} \right )\left ( \begin{matrix} j\\ k \end{matrix} \right )\frac{(-s)^{i-j}}{r^{m+i-j}}(-a)^{j-k}(b)^{-j}a_{n,j}, \\& t_{n,k}^{l}=(a+b)^{k}\sum _{i=k}^{l}\sum_{j=k}^{i} \left ( \begin{matrix} m+i-j-1\\ i-j \end{matrix} \right )\left ( \begin{matrix} j\\ k \end{matrix} \right ) \frac{(-s)^{i-j}}{r^{m+i-j}}(-a)^{j-k}(b)^{-j}a_{n,j} \end{aligned}$$

for all $n,k\in\mathbb{N}$.

By using Lemma 4.1, there are some immediate consequences with $t_{n,k}$ or $t_{n,k}^{l}$ in place of $a_{n,k}$ in Lemma 3.4 and Lemma 4.2.

### Theorem 4.3

$A\in(b_{c}^{a,b}(B^{(m)}):\ell_{p})$ *if and only if*

4.3 $$\begin{aligned}& \sup_{K\in\Gamma}\sum_{n} \biggl\vert \sum_{k\in K}t_{n,k}\biggr\vert ^{p}< \infty, \end{aligned}$$

4.4 $$\begin{aligned}& t_{n,k}\quad \textit{exists for each } k,n\in\mathbb{N}, \end{aligned}$$

4.5 $$\begin{aligned}& \sum_{k}t_{n,k}\quad \textit{converges for each } n\in\mathbb{N}, \end{aligned}$$

4.6 $$\begin{aligned}& \sup_{l\in\mathbb{N}}\sum_{k=0}^{l} \bigl\vert t_{n,k}^{l}\bigr\vert < \infty,\quad n\in \mathbb{N}. \end{aligned}$$

### Theorem 4.4

$A\in(b_{c}^{a,b}(B^{(m)}):\ell_{\infty})$ *if and only if* (4.4) *and* (4.6) *hold*, *and*

4.7 $$ \sup_{n\in\mathbb{N}}\sum_{k} \vert t_{n,k}\vert < \infty. $$

### Theorem 4.5

$A\in(b_{c}^{a,b}(B^{(m)}):c)$ *if and only if* (4.4), (4.6) *and* (4.7) *hold*, *and*

4.8 $$\begin{aligned}& \lim_{n\rightarrow\infty} t_{n,k} \quad\textit{exists for each } k\in\mathbb {N}, \end{aligned}$$

4.9 $$\begin{aligned}& \lim_{n\rightarrow\infty} \sum_{k}t_{n,k} \quad\textit{exists}. \end{aligned}$$

## Conclusion

By considering the definitions of the binomial matrix $B^{a,b}=(b^{a,b}_{n,k})$ and the difference operator, we introduce the sequence spaces $b_{0}^{a,b}(B^{(m)})$, $b_{c}^{a,b}(B^{(m)})$ and $b_{\infty}^{a,b}(B^{(m)})$. These spaces are the natural continuation of [18, 23–25]. Our results are the generalization of the matrix domain of the Euler matrix. In order to give full knowledge to the reader on related topics with applications and a possible line of further investigation, the e-book [29] is added to the list of references.
